# Malaria in Croatia: from eradication until today

**DOI:** 10.1186/1475-2875-11-S1-P135

**Published:** 2012-11-09

**Authors:** Mulić Rosanda

**Affiliations:** 1 School of Medicine, Department of Public Health, University of Split, Croatia

## Background

According to the World Health Organization, in Croatia malaria was officially eradicated in 1964. Since then a certain number of cases of imported malaria is registered every year, but this has seen a declining trend throughout the years.

## Materials and methods

A retrospective study about the incidence of imported malaria in Croatia in the period from 1987 to 2011 based on the official data of the Croatian National Institute of Public Health.

## Results

Thus at the beginning of the observed period there were 12 cases of imported malaria per year, whilst in the last five years that number dropped to a yearly average of 6. Between 1987 and 2011, Croatia recorded a total of 233 cases of imported malaria. The disease still most commonly affects seafarers and workers temporarily employed in malaria endemic countries. During 2011, of the 7 cases of imported malaria in Croatia, only 1 was a seafarer. It is very likely that the number of imported malaria cases is somewhat higher in seafarers but the disease often goes unnoticed among Croatian health services due to the fact that seafarers get treatment in ports around the world. The predominant causative agent for imported malaria is *Plasmodium falciparum*, which has been found in 61.8% (144/233) of patients. The disease is still mostly acquired during visits to Africa: 187 out of 234 (80.25%); visits to Asia account for a smaller portion (41/233; 17.6%), while South America has not been recorded as a source of imported malaria cases in the past 10 years.

Although almost all travellers and seafarers are advised to use chemoprophylaxis and ship management companies must provide chemoprophylaxis for their seafarers, irregular or non-existent application of chemoprophylaxis is the cause of imported malaria contraction. However, data on chemoprophylaxis should be considered with caution since these were obtained via patient polls and depend on their memory at that particular point in time.

## Conclusion

Malaria movements worldwide, the reoccurrence of autochthonous malaria cases in countries where the disease had been eradicated, the existence of malaria-transmitting mosquitos and a certain number of imported malaria cases in Croatia are all alarming facts. Therefore, health surveillance including a mandatory and adequate chemoprophylaxis for travellers to endemic areas remains a binding measure of public health care aimed at controlling malaria in Croatia.
